# A Comprehensive Review of Current Management Trends in Medial Compartment Arthritis of the Knee Joint

**DOI:** 10.7759/cureus.56666

**Published:** 2024-03-21

**Authors:** Kevin Kawde, Gajanan Pisulkar, Ankur Salwan, Adarsh Jayasoorya, Vivek H Jadawala, Shounak Taywade

**Affiliations:** 1 Orthopaedics, Jawaharlal Nehru Medical College, Datta Meghe Institute of Higher Education & Research, Wardha, IND

**Keywords:** patient outcomes, surgical procedures, non-surgical interventions, management trends, knee joint, medial compartment arthritis

## Abstract

Medial compartment arthritis of the knee joint presents a significant clinical challenge, with diverse management options ranging from nonsurgical interventions to various surgical procedures. This comprehensive review synthesizes current evidence on the management trends in medial compartment arthritis, highlighting both nonsurgical approaches such as physical therapy, pharmacological interventions, and intra-articular injections as well as surgical interventions, including arthroscopic debridement, high tibial osteotomy, and knee arthroplasty. Through a comparative analysis of efficacy, complication rates, and patient outcomes, this review underscores the importance of tailoring treatment strategies to individual patient characteristics and preferences. Furthermore, emerging techniques and technologies promise to advance the field, necessitating ongoing research efforts to refine treatment algorithms and establish standardized guidelines. By adopting a multidisciplinary approach and integrating evidence-based practices, clinicians can optimize the management of medial compartment arthritis and enhance patient care outcomes.

## Introduction and background

The knee joint is one of the largest and most complex joints in the human body, consisting of the articulation between the femur, tibia, and patella [1]. It is crucial for weight-bearing activities and locomotion, providing stability and mobility. The knee’s medial compartment refers to the joint’s inner aspect, where the femur and tibia meet [2]. Medial compartment arthritis, characterized by degeneration of the articular cartilage and underlying bone in the medial aspect of the knee joint, is a prevalent condition associated with significant pain and functional impairment [3]. Understanding this condition is vital due to its prevalence, impact on patient quality of life, and the variety of available management options [4].

This review aims to provide a comprehensive overview of current management trends in medial compartment arthritis of the knee joint. By examining both nonsurgical and surgical approaches and comparing their efficacy, complications, and patient outcomes, this review aims to offer insights into the optimal management strategies for patients with this condition. Additionally, it seeks to highlight emerging techniques and future directions in the field to guide clinical decision-making and improve patient care.

## Review

Nonsurgical management approaches

Physical Therapy and Exercise Regimens

Effectiveness of exercise therapy: Exercise therapy is a highly effective intervention for managing symptoms and impairments associated with hip and knee osteoarthritis (OA). Research indicates that engaging in exercise sessions three or more times weekly yields superior outcomes in individuals with OA [5]. For instance, a study comparing a therapeutic regimen combining manual physical therapy and supervised exercise with a placebo in patients with knee OA found significant improvements in the treatment group. These improvements included enhanced function, reduced pain and stiffness, and increased walking distance compared to the placebo group [6]. This highlights the substantial benefits of regular exercise therapy in alleviating symptoms and improving functional capacity in individuals with knee OA.

European Alliance of Associations for Rheumatology (EULAR) recommendations: The EULAR underscores nonpharmacological treatments, such as exercise, in managing knee OA-related pain and functional limitations. EULAR’s recommendations advocate for a comprehensive, multicomponent management plan that includes tailored exercise programs, maintaining a healthy weight, and incorporating behavior change techniques to promote a healthy lifestyle [7]. By emphasizing the importance of individualized exercise regimens and lifestyle modifications, these guidelines provide a holistic approach to managing knee OA and improving patients’ overall well-being.

Physical therapy benefits: Physical therapy offers a range of benefits for individuals with knee OA, making the condition more manageable through passive and active treatments. Passive treatments like cold and heat therapy can help alleviate pain and inflammation. Meanwhile, active treatments involve targeted exercises to enhance joint mobility, reduce pain and swelling, improve muscle strength, and optimize overall joint function [8]. By combining passive modalities with active exercises, physical therapy can address the multifaceted aspects of knee OA, leading to improved outcomes and enhanced quality of life for affected individuals.

Role of physical therapists: Physical therapists play a crucial role in managing knee OA, leveraging their expertise in movement to develop personalized treatment plans tailored to each patient’s specific needs and abilities. These individualized programs typically focus on strengthening the muscles surrounding the knee joint, improving range of motion, and enhancing overall mobility. By providing targeted interventions and ongoing support, physical therapists empower individuals with knee OA to actively participate in their care, ultimately facilitating better management of symptoms and optimizing functional outcomes [8].

Pharmacological Interventions

Oral medications: Oral medications play a significant role in managing knee OA, offering various options for pain relief and symptom management. Acetaminophen, recommended for mild to moderate pain associated with knee OA, is a first-line pharmacological therapy [9,10]. Non-steroidal anti-inflammatory drugs (NSAIDs), including diclofenac, ibuprofen, naproxen, and celecoxib, are commonly prescribed to alleviate pain and inflammation in knee OA patients [9,11,12]. Tramadol, an opioid analgesic, may provide modest benefits, particularly for older patients experiencing moderate to severe pain from knee OA [9]. These oral medications offer diverse options for pain management, allowing clinicians to tailor treatment regimens based on individual patient needs and preferences.

Intra-articular injections: Intra-articular injections represent another therapeutic option for managing knee OA symptoms, particularly for individuals who may not tolerate or respond adequately to oral medications. Corticosteroid injections are commonly administered to provide short-term symptomatic relief by reducing inflammation within the joint with minimal risk of systemic adverse effects [9]. Although their long-term efficacy remains uncertain, hyaluronic acid injections may offer short-term benefits by lubricating the joint and providing cushioning to alleviate pain and improve mobility [9]. These injections provide targeted relief directly to the affected joint, offering an alternative or adjunctive approach to oral medications for managing knee OA symptoms.

Complementary and alternative medicine (CAM): CAM modalities are increasingly recognized as adjunctive treatments for knee OA, offering additional options for symptom management and disease modification. Despite mixed evidence regarding their effectiveness as disease-modifying agents, glucosamine and chondroitin supplements are commonly used by individuals seeking to alleviate pain and improve joint function [9]. S-adenosylmethionine has shown efficacy comparable to NSAIDs in reducing pain and disability in knee OA patients, providing a potential alternative for those seeking nonpharmacological options [9]. Additionally, natural remedies such as ginger and turmeric have been suggested to provide clinical benefits in managing knee OA symptoms, offering further options for individuals exploring CAM approaches [9]. These complementary treatments offer a holistic approach to managing knee OA, addressing symptomatic relief and potential disease modification through nonconventional modalities.

Other pharmacological treatments: Beyond oral medications and intra-articular injections, other pharmacological treatments are available for managing knee arthritis symptoms. Topical therapies, including creams and gels, can be applied directly to the affected joint to provide localized relief while minimizing the systemic adverse effects of oral medications [9]. Although evidence regarding its efficacy remains weak, acupuncture may offer some benefit in alleviating pain and improving function in knee OA patients [9]. These additional pharmacological interventions broaden the spectrum of treatment options available for managing knee OA, offering diverse approaches to address the multifaceted nature of the condition and improve overall patient outcomes.

Surgical management options

Arthroscopic Debridement and Lavage

This procedure involves the introduction of saline solution into the knee joint to remove excess fluid and loose bodies, aiming to flush out and cleanse the joint space. Its goal is to alleviate arthritic knee inflammation or pain resulting from irritated synovial membrane growth [13]. Debridement involves smoothing the bone surface without further intervention or undertaking more invasive procedures like abrasion, meniscectomy, synovectomy, or osteotomy. It aims to eliminate fragments of joint material or degenerative tissue from the knee joint [13]. Research on the effectiveness of arthroscopic debridement and lavage for knee OA has yielded mixed results. Some studies suggest these procedures may not offer significant benefits compared to conservative treatments like physical therapy and medical management [14]. There is controversy surrounding the utility of simple lavage and debridement for older patients with established OA. Certain studies have failed to demonstrate the clear benefits of these procedures, prompting recommendations against routine arthroscopy with lavage and debridement in specific patient populations [14].

High Tibial Osteotomy (HTO)

HTO is one of the most effective approaches for managing medial compartment arthrosis, particularly in young, active patients with an intact lateral compartment. Its primary objective is to redirect the mechanical axis away from the degenerated area of the joint toward a relatively preserved region, ultimately yielding satisfactory long-term clinical and functional outcomes [15]. Typically, the ideal candidate for HTO is a middle-aged patient presenting with isolated medial OA, exhibiting a good range of motion, and lacking ligamentous instability. Precise indication, thorough preoperative planning, and careful selection of operative techniques are paramount to achieving favorable treatment outcomes [16].

Several techniques exist for performing HTO, with two common approaches being medial opening wedge osteotomy and closing wedge osteotomy. Medial opening wedge osteotomy is a frequently employed procedure that permits intraoperative correction in both the coronal and sagittal planes. However, it has certain drawbacks, such as elevated nonunion rates and potential leg lengthening [16]. In the context of the medial opening wedge HTO (MOWHTO), careful attention to soft tissue management and neurovascular protection is crucial to successfully treating medial compartment OA accompanied by varus malalignment [17]. These considerations are vital for optimizing surgical outcomes and minimizing postoperative complications associated with HTO procedures.

Unicompartmental Knee Arthroplasty (UKA)

UKA is a surgical intervention to manage OA localized to a single knee joint compartment. This procedure is typically considered when arthritis affects only one compartment, commonly observed in the medial aspect of the knee [18,19]. UKA can be revised to or with another UKA if the failure mode permits reconstruction with UKA components. In instances where disease progression extends to another compartment, additional UKA may be contemplated at the patellofemoral or remaining tibiofemoral joint. However, if these options prove impractical, primary total knee arthroplasty (TKA) may become necessary [18].

Studies suggest that UKA offers several advantages over TKA, including shorter hospital stays, reduced occurrence of serious medical complications, and quicker recovery periods [20]. Patients who undergo UKA tend to experience a faster return to sports activities than those undergoing TKA [20]. The procedure involves replacing the surface of the lower thigh bone with a new rounded metal surface and the surface of the upper shin bone with a flat metal surface, supplemented by a plastic insert between them to facilitate smooth joint movement [21]. UKA is less invasive than TKA, resulting in decreased pain, reduced bleeding, and a swifter recovery. It also allows for a greater range of motion, shorter in-patient stays, and easier revision to TKA if required [19]. However, it is important to note that there are contraindications to UKA, such as inflammatory arthritis involving the entire knee joint, significant obesity, severe deformities, knee stiffness, and other factors that could potentially impact the procedure’s success [19].

TKA

TKA involves replacing a damaged knee joint with a prosthesis constructed from metal and plastic components. The surgical procedure entails removing the compromised knee surfaces, affixing the prosthesis to the bone, and closing the incision using stitches or staples [22]. TKA is renowned for delivering dependable outcomes, including alleviating pain, enhancing quality of life, and restoring function for patients afflicted with end-stage degenerative knee OA [23,24]. Complications associated with TKA encompass stiffness, vascular injury, nerve palsy, metal hypersensitivity, and heterotopic ossification. Achieving optimal soft tissue balance during surgery is imperative for rectifying the valgus deformity [23,24]. Following TKA, patients typically undergo a hospital stay of several days and commence physical therapy shortly after surgery to regain muscle strength and range of motion. Continuous passive motion machines may assist in rehabilitation [22]. An interprofessional team is indispensable post-TKA to manage comorbidities effectively. This may entail dietary consultations, the implementation of pain management strategies, prophylaxis for deep vein thrombosis, and pharmacist guidance in selecting appropriate pain medications [23,24].

Emerging Techniques or Procedures

Medial compartmental knee replacement involves the utilization of a fixed-bearing prosthesis, such as the Miller-Galante implant, to address end-stage OA characterized by prevalent bone-on-bone contact [25]. Joint distraction has emerged as a viable treatment option for knee OA, particularly in cases reaching end-stage severity. This technique is designed to alleviate symptoms and potentially delay or even obviate the necessity for joint replacement surgery [26]. Partial knee replacement is garnering attention as a surgical approach tailored for late-stage medial compartment OA. This method aims to preserve healthy tissue while effectively addressing the affected compartment, offering a promising alternative to total knee replacement [27]. Innovative surgical treatments are being explored beyond traditional joint replacements and osteotomies, aiming to provide more tailored and efficacious solutions for patients grappling with medial compartment OA [28].

**Figure 1:**
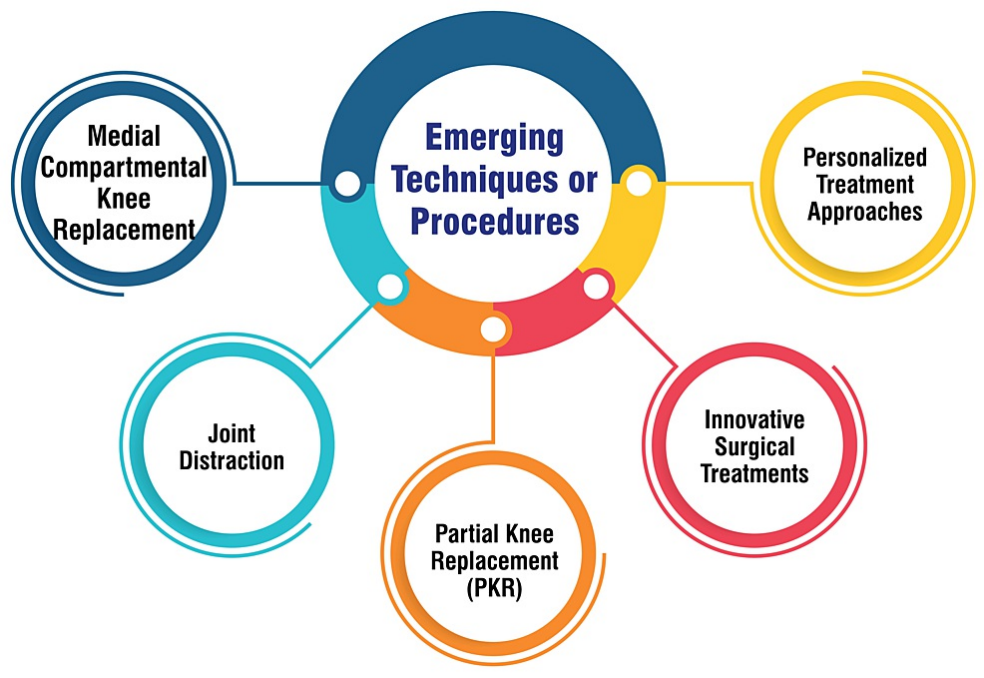
Emerging techniques or procedures Image credit: Kevin Kawde

The paradigm shift toward personalized medicine in orthopedics significantly influences the development of customized treatment plans. These plans consider individual patient characteristics and disease severity to optimize treatment outcomes [28]. The emergence of these innovative techniques and procedures reflects a growing trend toward more targeted, less invasive, and patient-centered approaches to managing medial compartment OA of the knee. By embracing innovation and individualized care, healthcare providers can offer patients a more comprehensive array of treatment options that alleviate symptoms and enhance quality of life and functional outcomes. Emerging techniques or procedures are shown in Figure 1.

Comparative analysis of management options

Efficacy

Pharmacologic interventions have been assessed for efficacy in treating primary knee OA using a network meta-analysis design. This method estimates relative effects among various treatments, offering valuable insights into their effectiveness and comparative performance [12]. Nonpharmacological approaches, such as lateral wedge insoles and valgus braces, have been studied for their efficacy in managing medial knee OA. While interventions like valgus bracing have demonstrated small to moderate effects on pain and function, recent meta-analyses suggest that lateral wedge insoles do not significantly improve pain outcomes [29].

The efficacy and cost-effectiveness of arthroscopic debridement for degenerative knee conditions have sparked debate. Evidence indicates that arthroscopic debridement may not confer superiority over sham surgery in treating degenerative knee conditions, prompting questions about its clinical effectiveness and cost-effectiveness relative to nonoperative treatments [30]. Current concepts in knee OA management emphasize nonoperative treatment options, underscoring the importance of a stepwise approach incorporating consensus recommendations for effective condition management [31,32]. This approach prioritizes conservative measures before considering surgical intervention, aligning with evolving perspectives on optimal treatment strategies for knee OA.

Complication Rates

Patients undergoing TKA for post-traumatic arthritis exhibit higher complication rates compared to those with OA [33]. Specifically, individuals with rheumatoid arthritis are more prone to infections within two years following surgery compared to patients with OA [34]. Moreover, patients undergoing bilateral TKA face an elevated risk of various complications, including stroke, blood loss, anemia, and the need for transfusion, in contrast to those undergoing unilateral TKA [35]. Despite the benefits of bilateral TKA in terms of reduced hospital stay and rehabilitation period, these patients encounter an increased likelihood of complications and readmission within 90 days post-surgery [35]. A comprehensive study comparing complications in patients undergoing bilateral versus unilateral TKA encompassed a substantial cohort matched for age, sex, race, and comorbidities, reflecting contemporary orthopedic practices observed between 2015 and 2020 [35]. Notably, complications such as pulmonary embolism, stroke, respiratory failure, blood loss-related anemia, and readmission within 90 days were more prevalent in the bilateral TKA group, despite concerted efforts to minimize blood loss during surgery [35].

Patient Satisfaction and Quality of Life Outcomes

Patient satisfaction and quality of life outcomes are pivotal in evaluating the effectiveness of treatments for knee OA. Numerous studies have identified factors influencing patient satisfaction, including treatment outcomes, room amenities, and food quality during hospital stays [36,37]. Research indicates that individuals undergoing primary total knee replacements generally express overall satisfaction with their knee-related quality of life and the care they receive. Factors such as joint function, pain relief, mental well-being, and vitality emerge as key determinants of satisfaction [37,38]. Moreover, hospitals are encouraged to prioritize identifying factors contributing to heightened patient satisfaction to enhance the quality of care rendered [37]. Additionally, it is essential to recognize that patient satisfaction is multifaceted and reflects patients’ perceptions of services. It furnishes valuable insights into meeting patient expectations and preferences, which are fundamental in today’s healthcare landscape characterized by patient-centered care and intensified competition among healthcare providers [37]. By comprehensively understanding and addressing the factors influencing patient satisfaction, healthcare institutions can cultivate a culture of excellence and continually strive to improve the patient experience.

Cost-Effectiveness

A study compared the cost-effectiveness of physical therapy and intra-articular glucocorticoid injections for knee OA. Results revealed that although physical therapy may entail higher initial costs, it is more cost-effective over the long term. This is evidenced by superior quality-adjusted life years (QALYs) and equivalent knee-related costs compared to glucocorticoid injections [39]. Furthermore, a systematic review delved into the cost-effectiveness of pharmacological therapy for managing OA. The review unveiled substantial heterogeneity among studies, with interventions such as NSAIDs, opioid analgesics, symptomatic slow-acting drugs for OA, and intra-articular injections exhibiting varying incremental cost-effectiveness ratios per QALY [40].

In surgical interventions, TKA and total hip arthroplasty emerged as cost-effective measures for addressing end-stage or severe knee and hip OA. While delaying these surgeries may yield short-term cost savings, it is deemed an unfavorable option from a long-term cost-effectiveness standpoint [41]. Given these findings, it is imperative to consider many factors, including initial costs, long-term outcomes, quality-of-life enhancements, and cost-effectiveness, when deliberating treatment options for osteoarthritic knee conditions. Physical therapy, pharmacological management, and surgical interventions each present distinct cost-effectiveness profiles that necessitate careful evaluation based on individual patient needs and preferences.

Considerations for clinical decision-making

Patient Characteristics and Preferences

**Figure 2:**
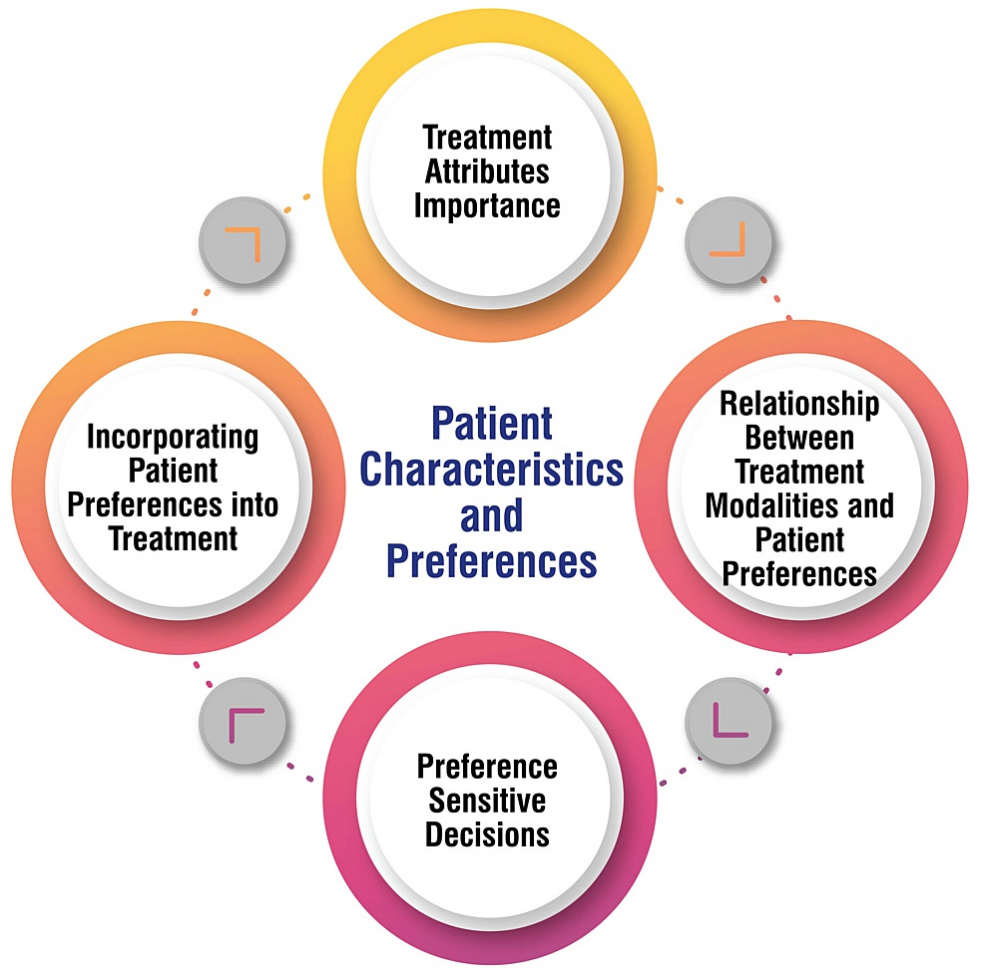
Patient characteristics and preferences Image credit: Kevin Kawde

Research has indicated that individuals with OA prioritize multiple treatment attributes, with outcomes such as the severity of joint pain, physical mobility, and the likelihood of experiencing severe side effects significantly influencing their preferences [42-44]. Understanding the intricate relationship between treatment modalities and patient preferences is paramount to managing OA. Patients’ decisions are shaped by factors such as the characteristics of medications, associated risks, benefits, and costs. Variations in potential adverse effects substantially influence treatment preferences [42,45]. Incorporating patient preferences into the treatment of OA is imperative for delivering personalized care. Studies have underscored that patients’ values regarding specific medication attributes vary, with considerations such as the risk of adverse effects and gastrointestinal ulcers prominently influencing their treatment choices [42,45]. Preference-sensitive decisions are relevant in the context of nonurgent, nonfatal diseases like OA. Patients’ willingness to accept risks and make trade-offs based on treatment attributes underscores the critical importance of eliciting and respecting patient preferences in the management of OA [45]. Patient characteristics and preferences are shown in Figure 2.

Disease Severity and Progression

MicroRNAs, small molecules within cells, have emerged as potential indicators of disease progression in knee OA. Recent research suggests that these molecules may serve as biomarkers capable of predicting the rate of progression from milder to more severe forms of the condition [46]. Their presence and activity levels in affected tissues have been linked to various pathological processes involved in OA development and progression, offering insight into the underlying molecular mechanisms driving the disease forward. Studies have identified a correlation between knee OA’s radiographic severity and disease progression trajectory. Interestingly, individuals with less severe initial radiographic findings may undergo more pronounced disease progression over time compared to those presenting with more advanced initial stages of OA [47]. This phenomenon underscores the complex nature of OA progression and challenges conventional assumptions about the relationship between disease severity and prognosis.

Knee OA exhibits considerable heterogeneity in its progression patterns, with some patients experiencing slow and gradual deterioration while others undergo rapid structural changes. Recent investigations have delineated distinct trajectories of structural disease progression, including early and late progression patterns [48]. These findings highlight the diverse clinical courses observed among individuals with knee OA and emphasize the need for personalized approaches to disease management. Several factors contribute to knee OA’s clinical and radiographic severity, influencing disease progression and its impact on affected individuals. Patient-reported outcomes, pain levels, changes in joint space width, and other clinical markers are crucial indicators for assessing disease severity and monitoring progression [49]. Understanding the multifaceted nature of these factors is essential for tailoring treatment strategies, implementing effective monitoring protocols, and ultimately enhancing outcomes in managing knee OA. By comprehensively addressing these disease severity and progression aspects, clinicians can optimize therapeutic interventions and provide personalized care to individuals with this prevalent orthopedic condition.

Future directions and challenges

Advancements in Surgical Techniques and Technology

MOWHTO represents a significant breakthrough in the surgical treatment of knee arthritis, emphasizing meticulous patient selection, comprehensive preoperative planning, and precise technical execution to maximize treatment efficacy [50]. This approach offers a tailored solution for addressing varus malalignment and cartilage damage, aiming to alleviate symptoms and preserve joint function. Minimally invasive surgery (MIS) techniques, including MIS for total knee replacement, have revolutionized surgical outcomes by employing smaller incisions and avoiding disruptive procedures like quadriceps tendon splitting and patellar eversion [51]. By minimizing tissue trauma, MIS facilitates quicker recovery and enhances short-term functional outcomes, ultimately improving patient satisfaction and postoperative rehabilitation.

**Figure 3:**
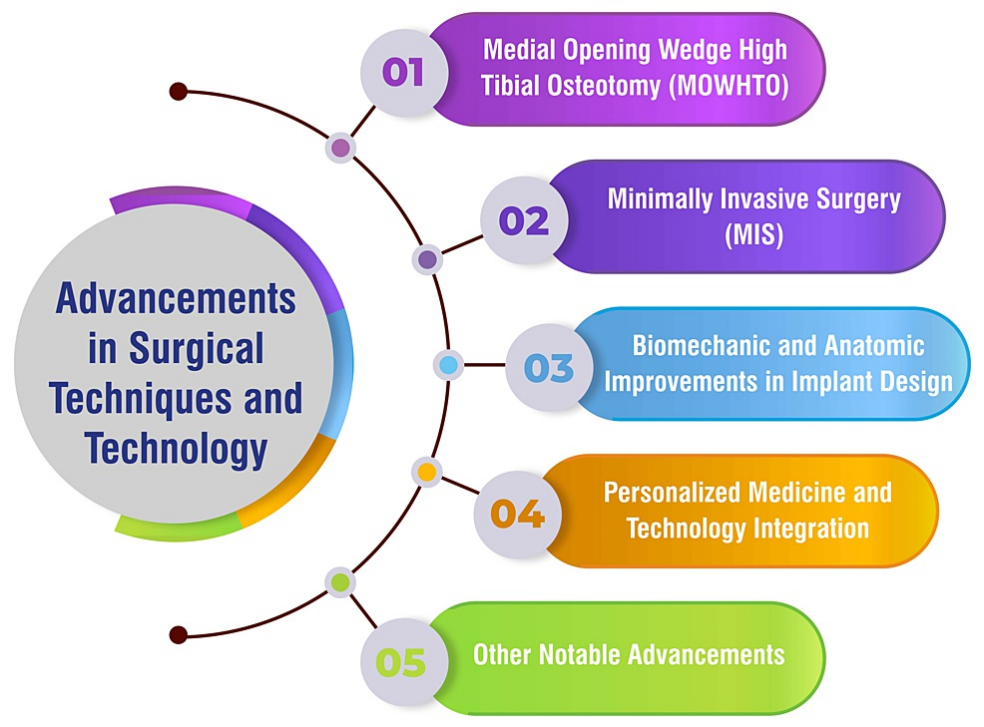
Advancements in surgical techniques and technology Image credit: Kevin Kawde

Advances in implant design have prioritized biomechanical and anatomical fidelity, striving to replicate the natural knee joint’s functionality more accurately [51]. Novel designs, inspired by the anatomy of the two cruciate ligaments and tailored to individual anatomical variations, promote better alignment and functionality following surgery, contributing to improved long-term outcomes. Integrating robotics, artificial intelligence (AI), and three-dimensional (3D) printing technologies into surgical practices and personalized treatment approaches informed by genomics and biomarkers heralds a promising era for enhancing surgical outcomes in knee arthritis management [52]. These innovative tools enable surgeons to optimize implant placement, tailor surgical procedures to individual patient anatomy, and predict treatment responses more accurately, ultimately improving patient outcomes and reducing the risk of complications. Noteworthy advancements such as UKA have demonstrated superior outcomes to traditional TKA, including lower joint awareness, reduced pain, improved function, and higher patient satisfaction [53]. These advancements underscore a paradigm shift toward precision-based, minimally invasive techniques, underscored by a commitment to personalized care and the refinement of implant designs, all aimed at optimizing patient outcomes in managing medial compartment arthritis of the knee joint. Advancements in surgical techniques and technology are shown in Figure 3.

Potential Areas for Research and Innovation

Nanotechnology-based drug delivery systems hold immense promise for revolutionizing OA treatment by leveraging nanoscale materials to deliver therapeutic agents directly into the joint capsule. This targeted approach allows for the concentration of drugs precisely at the site of pathology, potentially enhancing therapeutic efficacy while minimizing systemic side effects and reducing the risk of infection [54]. By harnessing the unique properties of nanoparticles, researchers aim to optimize drug delivery strategies, thereby improving treatment outcomes and advancing the standard of care for OA patients. Biological therapies and regenerative medicine represent cutting-edge approaches in OA management, focusing on harnessing the regenerative potential of biological agents such as platelet-rich plasma and stem cells. These therapies promise tissue regeneration and inflammation reduction, potentially providing innovative solutions for addressing the underlying pathology of knee OA [54]. By stimulating tissue repair mechanisms and modulating the inflammatory response, biological therapies hold the potential to mitigate disease progression and improve joint function, offering new avenues for OA treatment.

Personalized medicine and genomics are at the forefront of advancing OA management, offering the potential for tailored treatment strategies based on individual patient characteristics and genetic profiles. Genomics and biomarker research advances enable clinicians to identify patients most likely to benefit from specific treatments, optimizing therapeutic outcomes and revolutionizing knee arthritis management [54]. By leveraging personalized medicine approaches, clinicians can deliver more targeted and effective treatments, thereby improving patient outcomes and enhancing the overall quality of care for OA patients. Technology integration in surgical procedures, including the adoption of robotics, AI, and 3D printing, holds promise for enhancing precision and optimizing surgical outcomes in knee arthritis management. These advanced technologies enable surgeons to perform procedures more accurately and efficiently, improving patient outcomes and reducing complication rates [54]. By integrating these technologies into surgical practice, clinicians can advance the standard of care for OA patients, paving the way for innovative treatment approaches and improved long-term outcomes.

**Figure 4:**
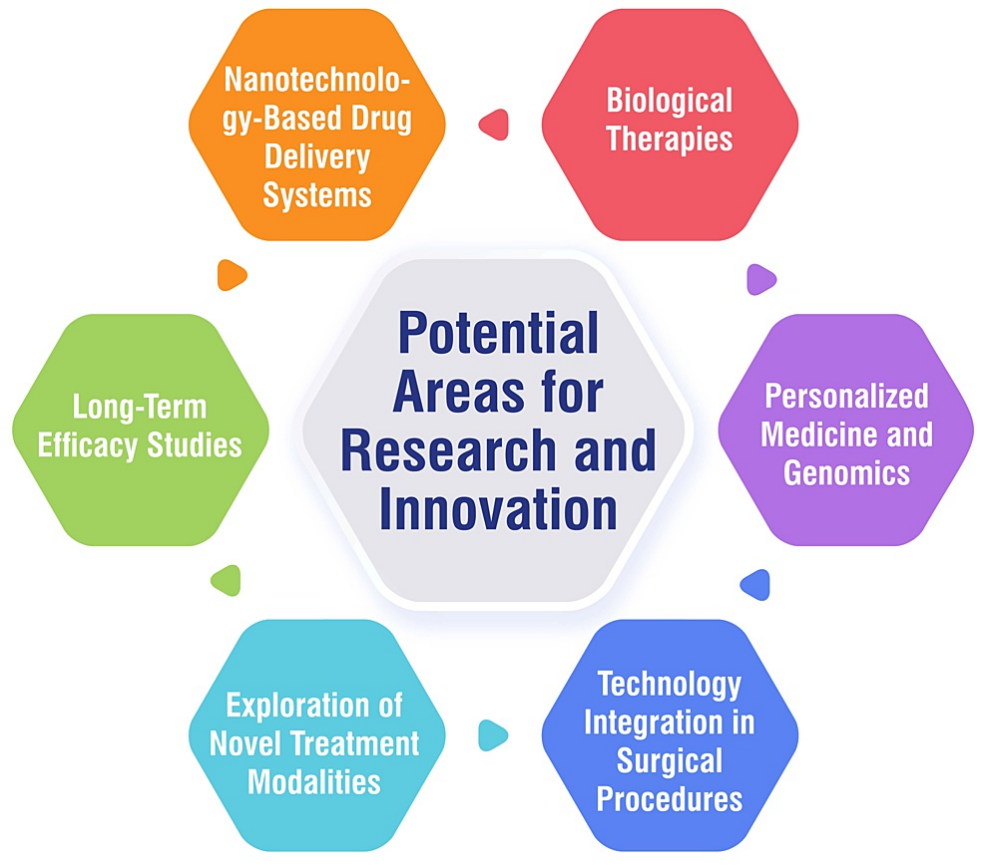
Potential areas for research and innovation Image credit: Kevin Kawde

Exploration of novel treatment modalities, such as genicular nerve radiofrequency ablation and arthroscopic cartilage regeneration facilitating procedures, offers new avenues for managing knee OA effectively [55]. These innovative treatments target specific pain pathways and promote tissue regeneration, providing alternative options for patients who may not respond to traditional therapies. By exploring novel treatment modalities, researchers aim to expand the arsenal of treatment options available for OA patients, ultimately improving outcomes and quality of life. Long-term efficacy studies are essential for validating emerging treatments’ clinical benefits and safety profiles, such as nanotechnology-based drug delivery systems and biological therapies [55]. By conducting extensive long-term studies, researchers can assess the durability of treatment effects, monitor for potential adverse events, and ensure that new therapies meet the highest safety and efficacy standards. These studies are critical for informing clinical practice guidelines and guiding treatment decisions, ultimately improving patient care and outcomes in OA management. Potential areas for research and innovation are shown in Figure 4.

## Conclusions

This review provides a comprehensive understanding of the management trends in medial compartment arthritis of the knee joint. Nonsurgical interventions like physical therapy and pharmacological treatments offer symptomatic relief. They may slow disease progression, while surgical options such as arthroscopic debridement, HTO, and knee arthroplasty present effective long-term solutions, each with their own indications and considerations. Patient-specific factors, including disease severity and patient preferences, should guide treatment decisions, emphasizing the importance of a personalized approach. Furthermore, ongoing research efforts should aim to refine treatment algorithms, explore novel interventions, and establish standardized guidelines to optimize patient outcomes and ensure consistent, high-quality care delivery. By integrating emerging techniques and fostering collaboration among healthcare professionals, clinicians can enhance the management of medial compartment arthritis and improve the overall well-being of affected individuals.
